# Prevalence of congenital septal defects among congenital heart defect patients in East Africa: A systematic review and meta-analysis

**DOI:** 10.1371/journal.pone.0250006

**Published:** 2021-04-22

**Authors:** Yossef Teshome Zikarg, Chalachew Tiruneh Yirdaw, Teshome Gebremeskel Aragie

**Affiliations:** 1 College of Health Sciences, School of Medicine, Department of Anatomy, Addis Ababa University, Addis Ababa, Ethiopia; 2 College of Health Sciences, Department of Anatomy, Wollo University, Dessie, Ethiopia; 3 College of Health Sciences, Department of Anatomy, Woldia University, Woldia, Ethiopia; Zagazig University, EGYPT

## Abstract

**Introduction:**

Congenital heart defects (CHDs) are the most common congenital defects and accounts for nearly one-third of all major congenital anomalies. It is the leading causes of birth defect-associated morbidity, mortality, and medical expenditures. Of all CHD types, ventricular septal defect (VSD) and atrial septal defect (ASD) accounted 51% of cases with an increasing trend over time.

**Objective:**

The aim of this review is to estimate the pooled prevalence of ventricular septal defect and congenital atrial septal defect among congenital heart diseases patients in East African context.

**Methods:**

Using PRISMA guideline, we systematically reviewed and meta-analyzed studies that examined the prevalence of Ventricular septal defect and atrial septal defect in East Africa, from Medline (PubMed), Cochrane Library, HINARI, and Google Scholar. A weighted inverse variance random-effects model was used to estimate the pooled prevalence of ventricular septal defect and atrial septal defect.

**Results:**

A total of 2323 studies were identified; 1301 from PubMed, 12 from Cochrane Library, 1010 from Google Scholar and 22 from other sources. The pooled prevalence of ventricular septal defect and atrial septal defect in East Africa was found to be 29.92% (95% CI; 26.12–33.72; I2 = 89.2%; p<0.001), and 10.36% (95% CI; 8.05–12.68; I2 = 89.5%; p<0.001) respectively.

**Conclusions and future implications:**

Based on this review, the pooled prevalence of VSD and ASD is still high and alarming; this signifies that the emphasis given for congenital heart defect in East African countries is limited. Special attention and efforts should be applied for early detection to prevent serious complications and for a better prognosis of all forms of CHD. A screening program for CHD should be instituted during the perinatal period. Furthermore, early referral of suspected cases of congenital cardiac anomalies is mandatory for better management till the establishment of cardiac centers in different regions of the continent.

## Introduction

Congenital heart defect (CHD) is typically defined as a structural abnormality of the heart and/or great vessels present at birth [1]. It is one of the most common congenital defects and accounts for nearly one-third of all major congenital anomalies [2] and it is the leading cause of birth defects-associated morbidity, mortality, and medical expenditures [3]. Of all CHD types ventricular septal defect (VSD) and atrial septal defect (ASD) accounted for 51% with an increasing trend over time [1, 4].

A review of [5] Hoffman and Kaplen (2002) revealed that, the incidence of all forms of CHD is 75/1,000 live births; of them moderate and severe forms of CHD is about 6/1,000 live births. Although most cases of CHD cured spontaneously without any intervention, the overall incidence that require expert cardiologic care ranges from 2.5 to 13/1,000 live births. Moreover, in neonates with severe form of CHD, mortality is high in low and lower-middle-income countries like sub-Saharan African countries than those living in high-income countries. Nine-tenths of the world’s babies born with CHD live in locations with little to no care where mortality remains high [6].

In Africa, approximately 50 million live children are born every year and an estimated 500,000 children have significant CHD that require an expert for cardiological care. However, they have little or no access to treatment of any kind. About half of cases deceased within a few years of birth and one-third of them within the first month of life [7, 8]. Additionally, a major proportion of older children and adults with CHD in sub-Saharan Africa survived for longer times are debilitated and live in under chronic illness [8].

According to CHD data of European Commission from 2000–2018 revealed that, congenital ventricular and atrial septal defects accounted (45.6%) and (23.54%) respectively; which is more than two-third of all types of CHD [2]. Similarly, these forms of CHDs have higher proportion in different regions of Africa. For instance, ventricular and atrial septal defects in Cameroon 38.8% and 2.8% [9], Djibouti 28% and 0.13% [10] in Ethiopia 27%–62% and 6.4%–23.7% [11–17], in Kenya 16.1% -18.7% and 4.7%–6.2% [18, 19] in Nigeria 28%–31% and 21%–29% [20, 21], in Sudan 29%–34.5% and 6% -17% [22, 23], in Tanzania 10%–34.6% and 3.7%–10% [24–26] and in Uganda 27.2% and 9.4% [27] respectively.

Despite, the availability of these data in a scattered form a pooled data is missed particularly in the Eastern regions of Africa. Previously, two systematic reviews and meta-analyses have shown the magnitude of CHD across the world and shown a significant heterogeneity between different continents with a suggestion prevalence of CHD is rising globally (9 per 1000 live births). However, in Africa (1.9 per 1,000 live births) the prevalence of CHD is significantly lower than other regions of the world [4]. The possibility that, this low birth prevalence might be due to missing of fully representative data in the continent. It might be also due to a few number of articles included in their reviews from the region; evidenced by only 6 articles included from the entire regions of African continent with no article from the Eastern regions of Africa [1, 4]. Therefore, the present systematic review and Meta-analysis is designed to estimate the pooled prevalence of congenital ventricular and atrial septal defects among congenital heart disease patients in Easter African context from January 2000-October 2020.

## Methods

### Review question

The review questions of this systematic review and meta-analyses was:

What is the pooled prevalence of congenital ventricular septal defect and congenital atrial septal defect among congenital heart disease patients in East African context?

### Study selection and screening

The retrieved studies were exported to Endnote version 8 reference managers to remove duplicate studies. Two investigators (YT and TG) independently screened the selected studies using the article’s title and abstracts before retrieval of full-text papers. We used pre-specified inclusion criteria to further screen the full-text articles. Disagreements were discussed during a consensus meeting with another reviewer (CT) for the final selection of studies to be included in the systematic review and meta-analyses.

### Inclusion and exclusion criteria

In this systematic review and meta-analyses, we have included cross-sectional and cohort studies of populations residing in all East African countries reporting at least the prevalence of VSD and/or ASD with enough data to compute the estimates, regardless of stillbirth. Studies published in the English language from January 2000—October 2020 were included. Citations without abstract and/or full-text, anonymous reports, editorials, letters, commentaries, reviews, and qualitative studies were excluded from the analyses. Besides, Studies conducted among populations of African origin residing outside Africa, studies without echocardiographic confirmation of CHD (e.g. suspected but non-confirmed CHD) were excluded from this review.

### Search strategy

This review identified published and unpublished studies that provide data on the prevalence of CHD (ASD and VSD) in the context of Eastern Africa. Relevant studies were identified through a literature search of Medline (PubMed), EMBASE, HINARI, Google scholar, Science Direct, Cochrane Library and other sources. A snowball searching of the references of relevant papers for linked articles was also performed. Using the key terms, the following search map was applied: (prevalence OR magnitude OR incidence OR pattern) AND (children [MeSH Terms] OR (child [MeSH Terms]) OR (infant [MeSH Terms]) AND (Birth defect [MeSH Terms] OR (Congenital Anomaly [MeSH Terms] OR (Congenital heart defect [MeSH Terms] OR congenital heart disease [MeSH Terms] OR Atrial Septal Defect [MeSH Terms] OR (Ventricular Septal defect) [MeSH Terms] AND “each country in Eastern Africa region”. These search terms were further paired with the names of each East African country. On both Cochran Library and Google scholar, a build-in text search was used on the advanced search section of the sources. All the literatures accessible from January 2000-October, 2020 were included in the systematic review and meta-analyses.

### Quality assessment

To evaluate the quality of the studies included in this review, three authors independently appraised the quality of the studies by using the Joanna Briggs Institute (JBI) quality appraisal checklist [28] and discrepancies were resolved by discussion. Studies were considered as low risk or good quality when it scored 4 and above for all designs (cross-sectional and cohort) whereas the studies scored 3 and below were considered as high risk or poor quality [28].

### Data extraction

The authors developed a data extraction form on the excel sheet considering the country, year of publication, study design, and prevalence of ventricular septal defect and atrial septal defect reported. The data extraction sheet was piloted using 4 papers randomly, and it was adjusted after piloted the template. Two of the authors extracted the data using the extraction form in collaboration and any discrepancy resolved through discussions with a third reviewer when required. The third author checked the correctness of the data independently. The mistyping of data was resolved through crosschecking with the included papers.

### Synthesis of results

The authors exported the data to STATA 14 for analysis after it was extracted in the excel sheet. We pooled the overall prevalence estimates of the ventricular septal defect and atrial septal defect by a random effect meta-analyses model. We examined the heterogeneity of effect size using Q statistic and the I2 statistics. In this study, the I2 statistic value of zero indicates true homogeneity, whereas the values 25, 50, and 75% represented low, moderate, and high heterogeneity, respectively [29]. Subgroup analysis was done by the study country, study design, and year of publication. Sensitivity analysis was employed to examine the effect of a single study on the overall estimation. Publication bias was checked by funnel plot and more objectively through Egger’s regression test.

## Results

**Fig 1:**
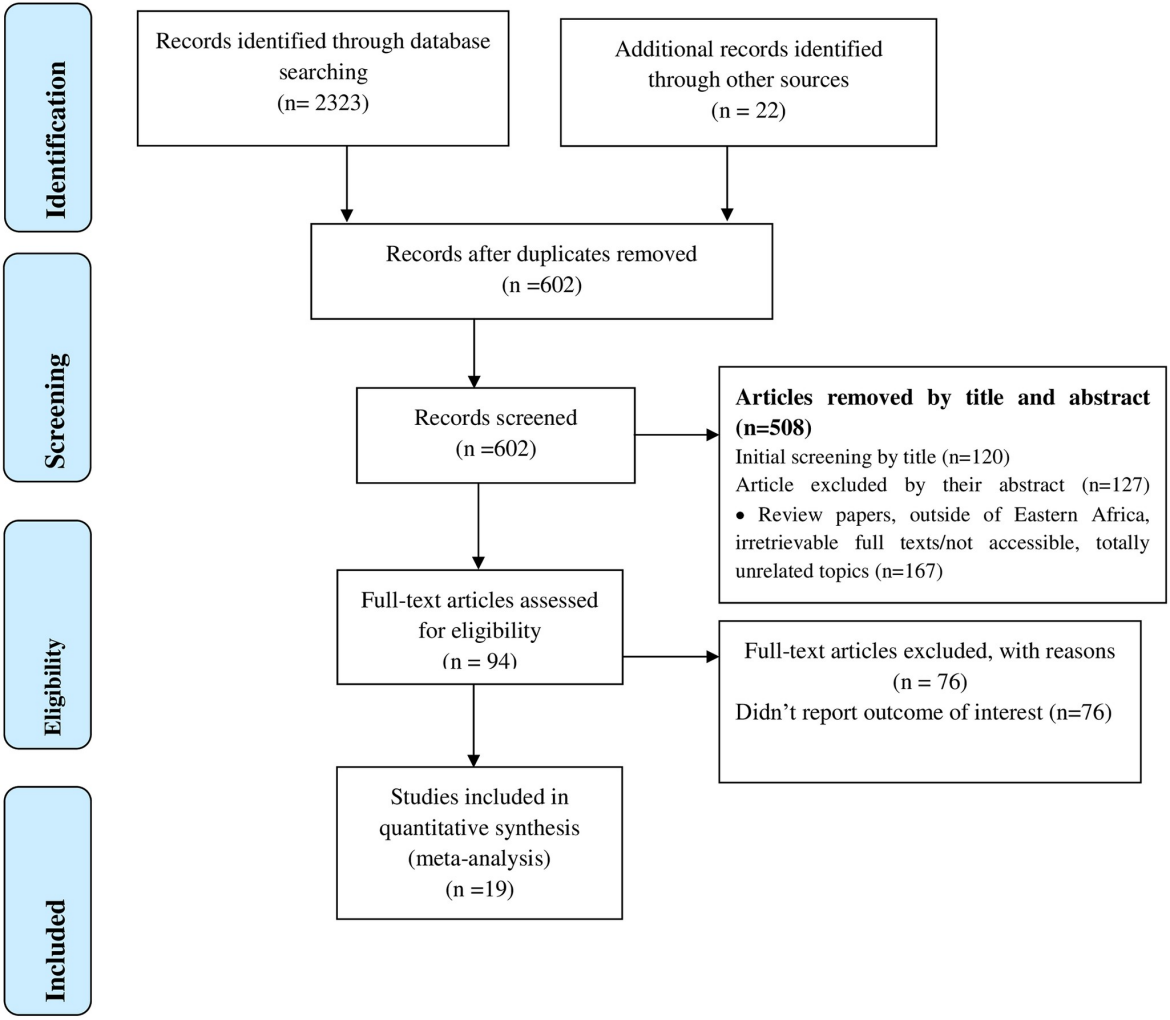
PRISMA–adapted flow diagram showed the results of the search and reasons for exclusion [33].

Using a pre-specified protocol, a total of 2323 studies was identified; 1301 from PubMed, 12 from Cochrane Library, 1010 from Google Scholar, and 22 from other sources. After duplication was removed, a total of 630 articles remained (1715 removed by duplication). Finally, 94 full-text studies were reviewed and 18 articles with 9109 patients have met the inclusion criteria and were selected for the analysis (Fig 1).

### Characteristics of included studies

A total of 18 studies were included in this systematic review and meta-analyses, encompassing 9109 patients [10–19, 22–27, 30, 31]. Of them 8 studies were done in Ethiopia [11–17, 30], 2 in Kenya [18, 19], while 3 were in Sudan [22, 23, 31], 1 in Djibouti [10], 1 in Uganda [27], and 3 in Tanzania [24–26]. Based on the study design used 15 studies were done by cross-sectional study design [11–17, 19, 22–25, 27, 30, 31] and other 3 studies were conducted by cohort study design [10, 18, 26]. 12/18 (66.6%) were published with in the year 2011–2020 and the remaining 6/18 (33.4%) were published with in the year 2000–2010. The total number of participants in the included studies ranged from 21 [17] to 3526 [27] (Table 1).

**Table 1:** Distribution of included studies on the magnitude of congenital septal defects among congenital heart defect patients in East Africa, from January 2000- October 2020.

Author Name	Publication year	Country	Study design	No of CHD cases	ASD	VSD	Method of diagnosis	References
Afework et al.	2020	Ethiopia	Cross sectional	1111	10.44		echocardiography	[14]
Moges	2008	Ethiopia	Cross sectional	21	9.5	42.8	echocardiography	[17]
Seyoum et al.	2018	Ethiopia	Cross sectional	97	23.70	30.9	echocardiography	[11]
Mehadi et al.	2006	Ethiopia	Cross sectional	177	13	27.1	echocardiography	[15]
Yadeta et al.	2017	Ethiopia	Cross sectional	1115	21.3	34.9	echocardiography	[16]
Haddish et al.	2019	Ethiopia	Cross sectional	160	16.9	36.8	echocardiography	[12]
Massoure et al.	2013	Djibuti	Cohort	27	13	37.00	echocardiography	[10]
Ibrahim et al.	2012	Sudan	Cross sectional	143	7	34.3	echocardiography	[23]
Namuyonga et al.	2020	Uganda	Cross sectional	3526	9.4	27.2	echocardiography	[27]
Zuechner et al.	2019	Tanzania	Cross sectional	1371	7.1	26.1	echocardiography	[25]
Elbadri et al.	2003	Sudan	Cross sectional	100	22	27	echocardiography	[22]
Elbadri et al.	2003	Sudan	Cross sectional	100	12	42	echocardiography	[22]
Sulafa et al.	2007	Kenya	Cohort	378	6.2	16.1	echocardiography	[18]
Raphael et al.	2018	Tanzania	Cohort	20	10	10	echocardiography	[26]
Shija	2013	Tanzania	Cross sectional	107	3.7	34.6	echocardiography	[24]
Gebi et al.	2013	Ethiopia	Cross sectional	157	6.4	26.8	echocardiography	[13]
Shamsedien	2013	Sudan	Cross sectional	170	6	29	echocardiography	[31]
Awori et al.	2013	Kenya	Cross sectional	214	4.7	18.7	echocardiography	[19]
Malede et al.	2006	Ethiopia	Cross sectional	76	6.6	62	echocardiography	[30]

### Meta-analysis

#### Ventricular septal defect

**Fig 2:**
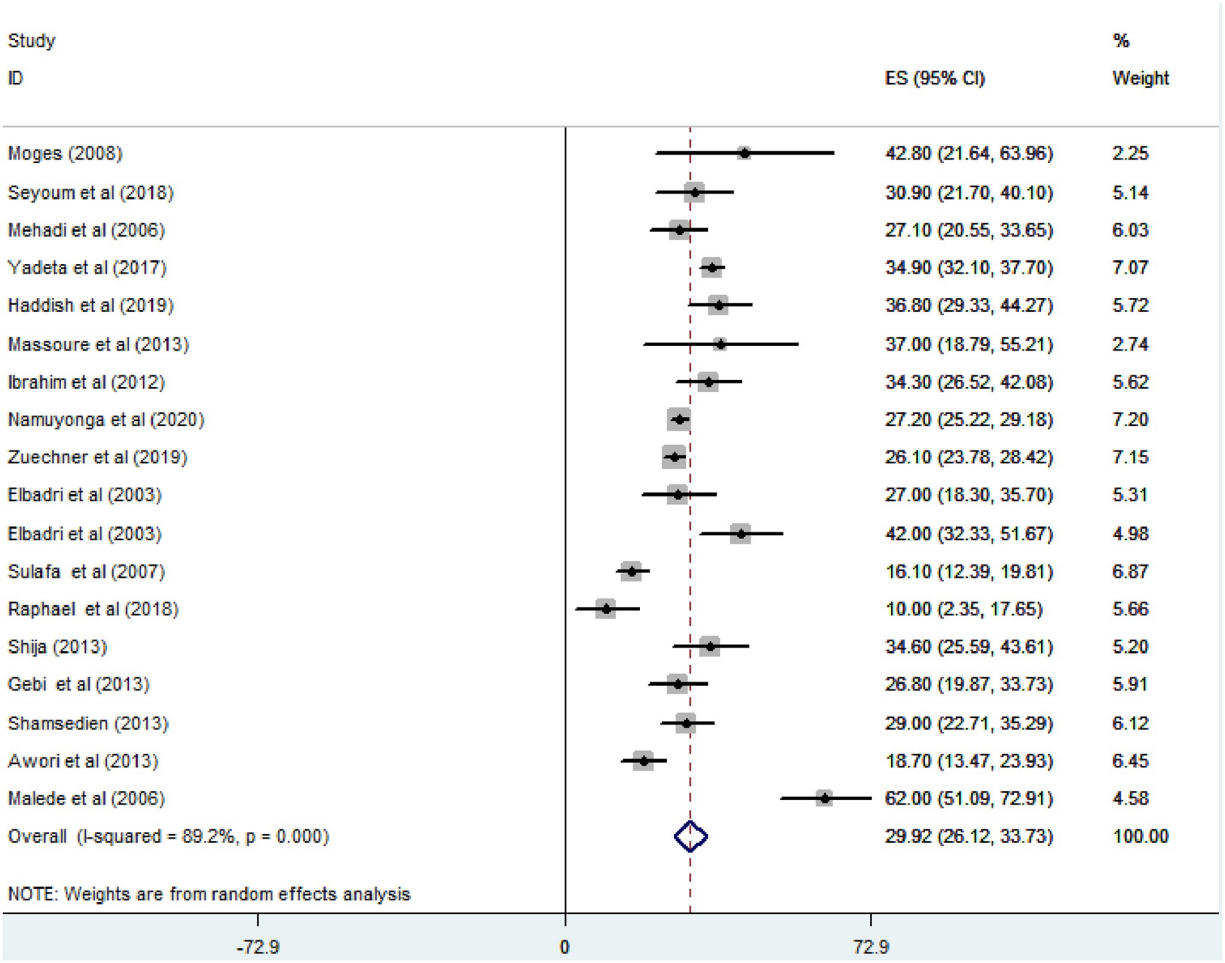
Forest plot showing the pooled magnitude of ventricular septal defect among congenital heart defect patients in East Africa, from January 2000-October 2020.

*Prevalence of ventricular septal defect among congenital heart defect patients*. Except one, all of the studies (n = 18) have reported the prevalence of ventricular septal defect among congenital heart defect patients. The prevalence of VSD was ranged from 10% [26] to 62% [30]. The random-effects model analysis from those studies revealed that the pooled prevalence of VSD among CHD patients in East Africa was found to be 29.92% (95% CI; 26.12–33.72; I2 = 89.2%; p<0.001) (Fig 2).

**Fig 3:**
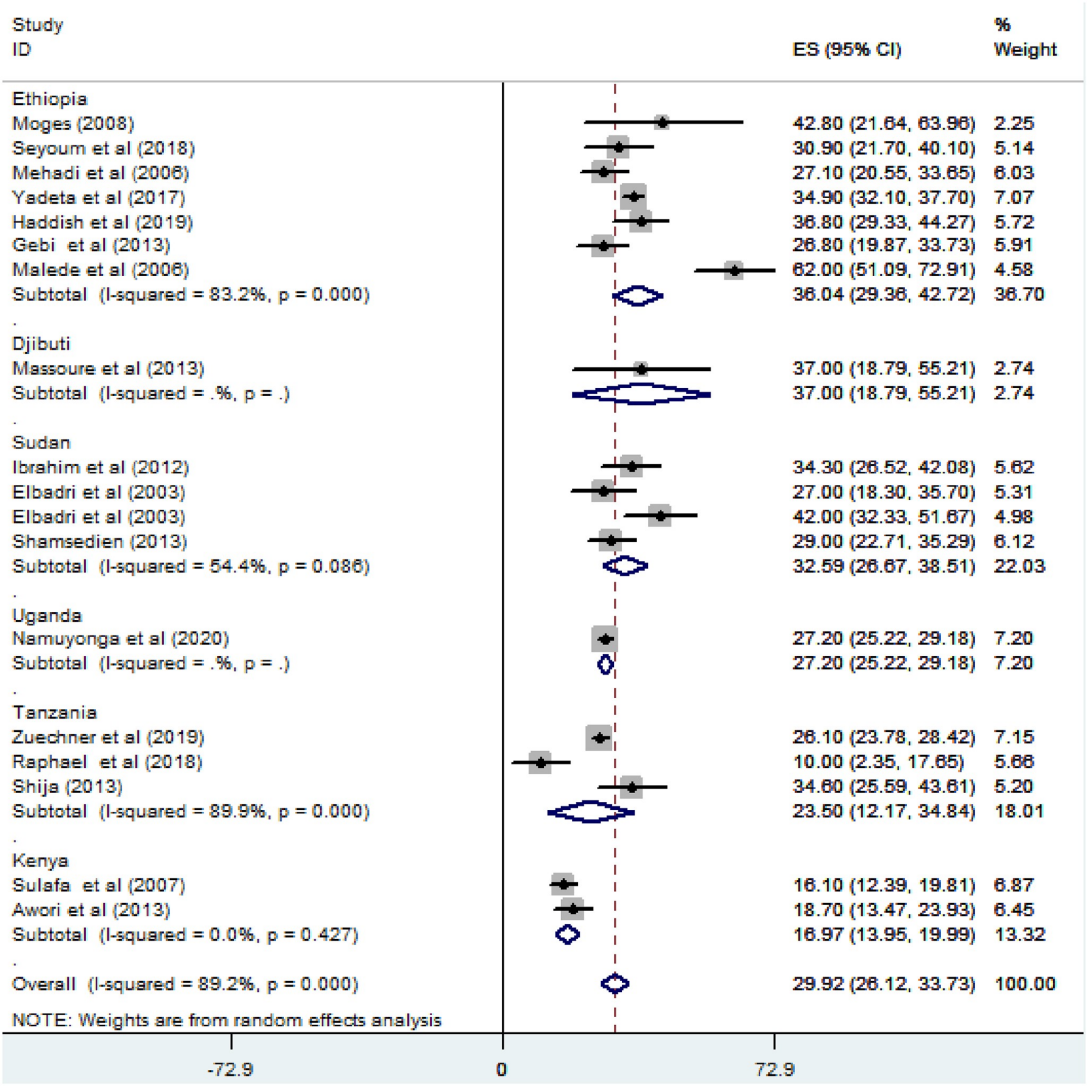
Forest plot showing the subgroup analysis of the pooled magnitude of ventricular septal defect among congenital heart defect patients based on the country in East Africa, from January 2000-October 2020.

*Subgroup analysis for the prevalence of Ventricular Septal Defect (VSD) among congenital heart defect patients in Eastern Africa*. The subgroup analysis was done through stratified by country, study design, and year of publication. Based on this, the prevalence of VSD among congenital cardiac patients was found to be 36.04% (95% CI; 29.36–42.72%) in Ethiopia, 37% (95% CI: 18.79–55.21%) in Djibouti, and 32.59% (95% CI: 26.67–38.59%) in Sudan (Fig 3).

**Fig 4:**
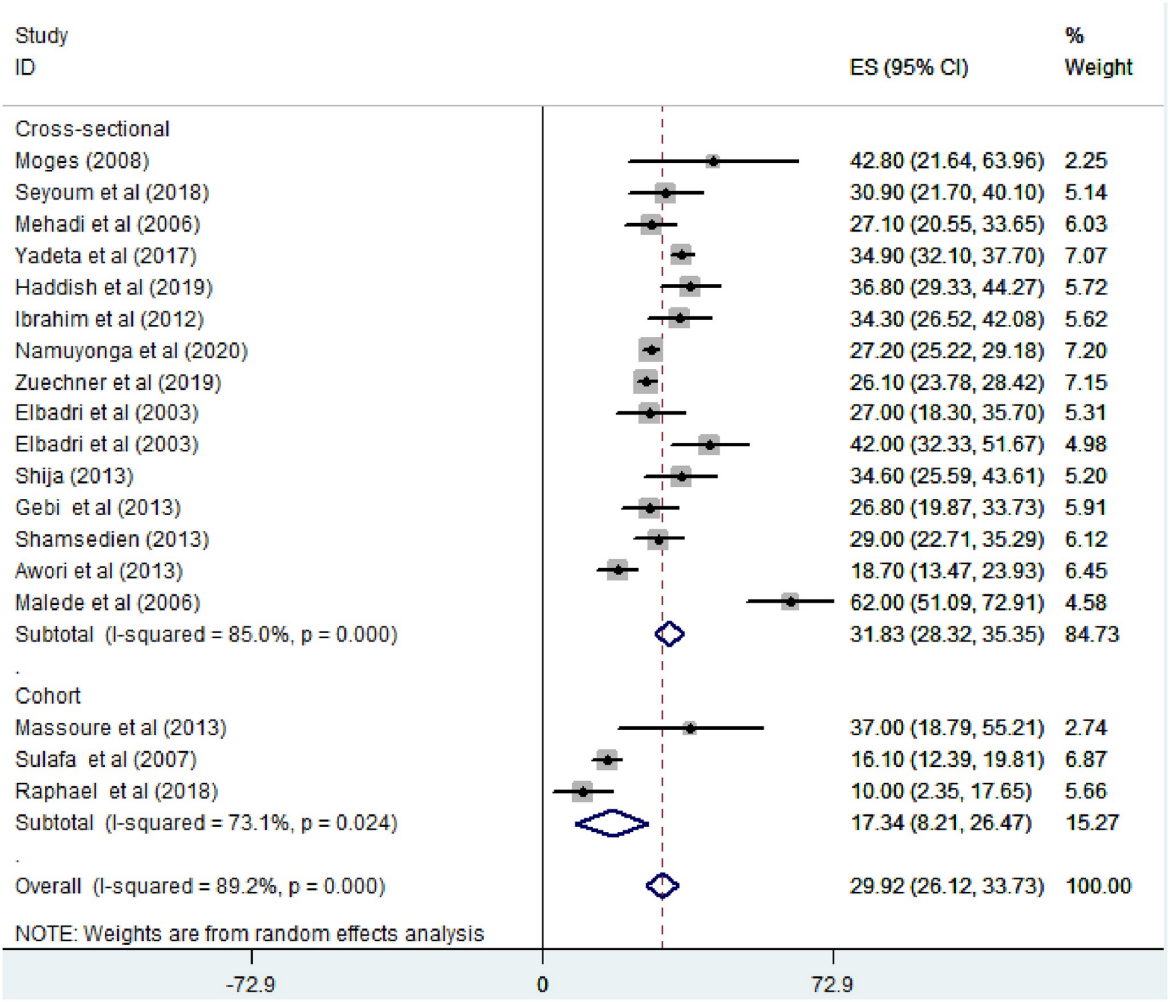
Forest plot showing subgroup analysis of the pooled magnitude of ventricular septal defect among congenital heart defect patients by study design in East Africa, from January 2000-October 2020.

**Fig 5:**
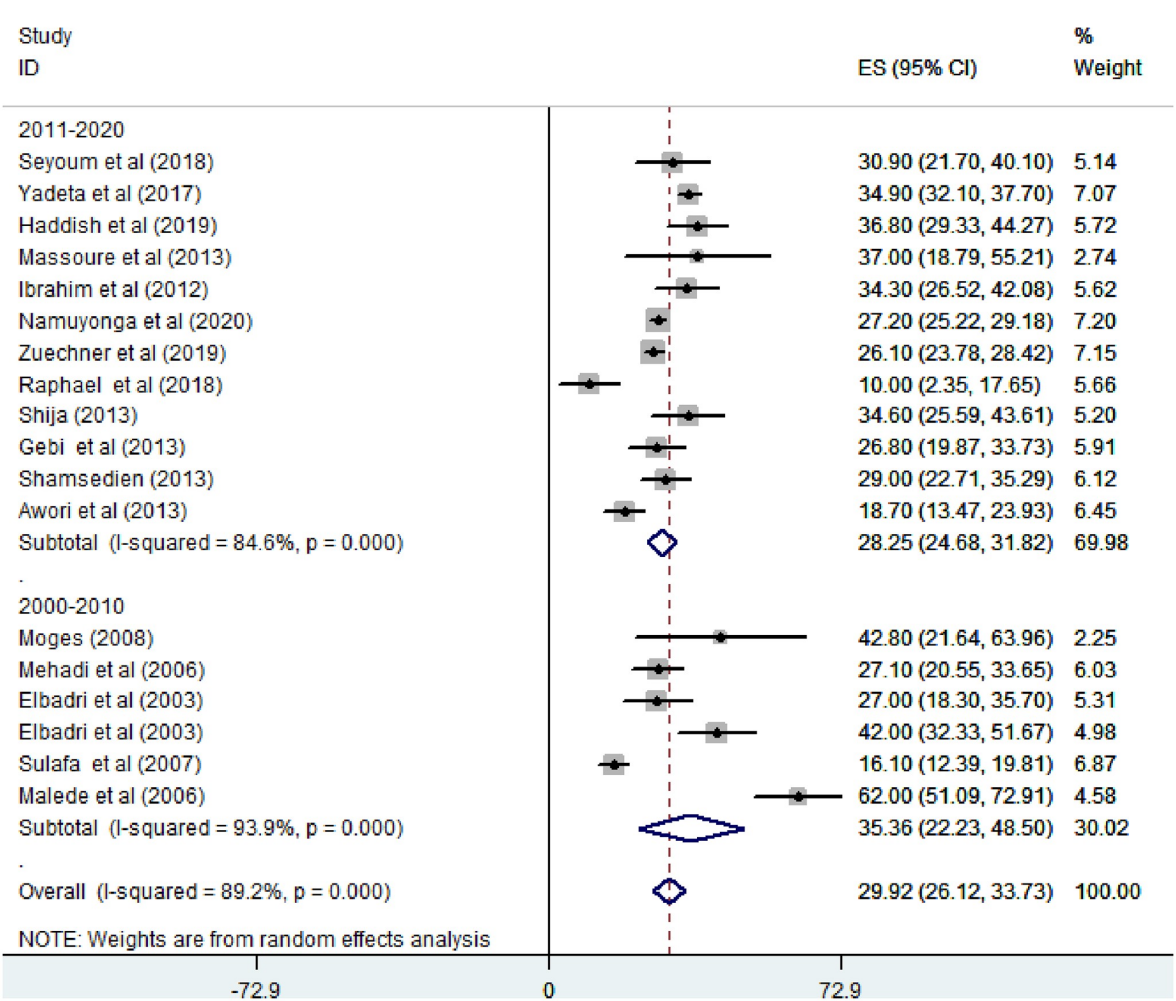
Forest plot showing subgroup analysis of the pooled magnitude of ventricular septal defect among congenital heart defect patients by year of publication in East Africa, from January 2000-October 2020.

Based on the study design, the prevalence of VSD among CHD patients was found to be 31.82% in cross-sectional studies and 17.34% (95% CI: 8.21–26.47%) in cohort studies (Fig 4). Regarding year of publication, the prevalence of VSD among CHD patients was found to be 35.36% (95% CI: 22.23–48.50%) where, studies conducted from January 2000—October 2010 while it was 28.25% (95% CI: 24.68–31.82%) where, studies conducted from 2011–2020 (Fig 5).

### Heterogeneity

#### Sensitivity analysis for ventricular septal defect

We employed a leave-one-out sensitivity analysis to identify the influence of individual studies on the pooled prevalence of ventricular septal defect in Eastern Africa. The results of this sensitivity analysis showed that our findings were not dependent on a single study. The pooled estimated prevalence of VSD varied between 28.22 [30] and 30.79 [18] after the deletion of a single study (S1 Fig).

### Publication bias

**Fig 6:**
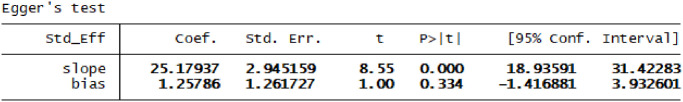
Publication bias on the magnitude of ventricular septal defect among congenital heart defect patients in East Africa from January 2000-October, 2020.

A funnel plot showed symmetrical distribution. The Egger’s regression test-value was 0.334, which indicated that, the absence of publication bias (Fig 6 and S2 Fig).

#### Atrial septal defect

**Fig 7:**
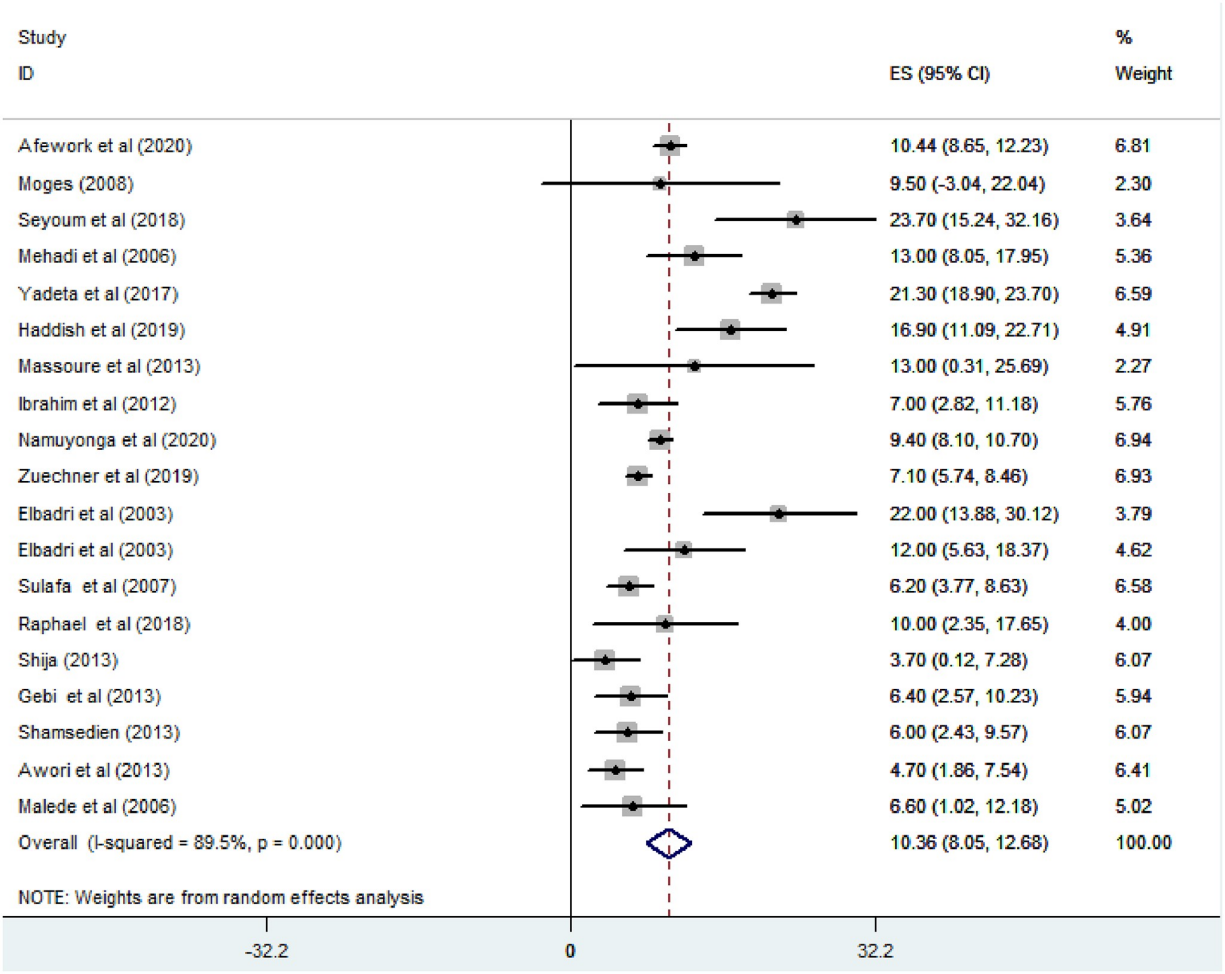
Forest plot showing the pooled prevalence of atrial septal defect among congenital heart defect patients in East Africa, from January 2000-October 2020.

*Prevalence of atrial septal defect among congenital heart defect patients*. All of the studies included (n = 19) have reported the prevalence of ASD among CHD patients. The prevalence of ASD was ranged from 3.7% [24] to 23.7% [11]. The random-effects model analysis from those studies revealed that the pooled prevalence of ASD among CHD patients in East Africa was found to be 10.36% (95% CI; 8.05–12.68; I2 = 89.5%; p<0.001) [Fig 7].

**Fig 8:**
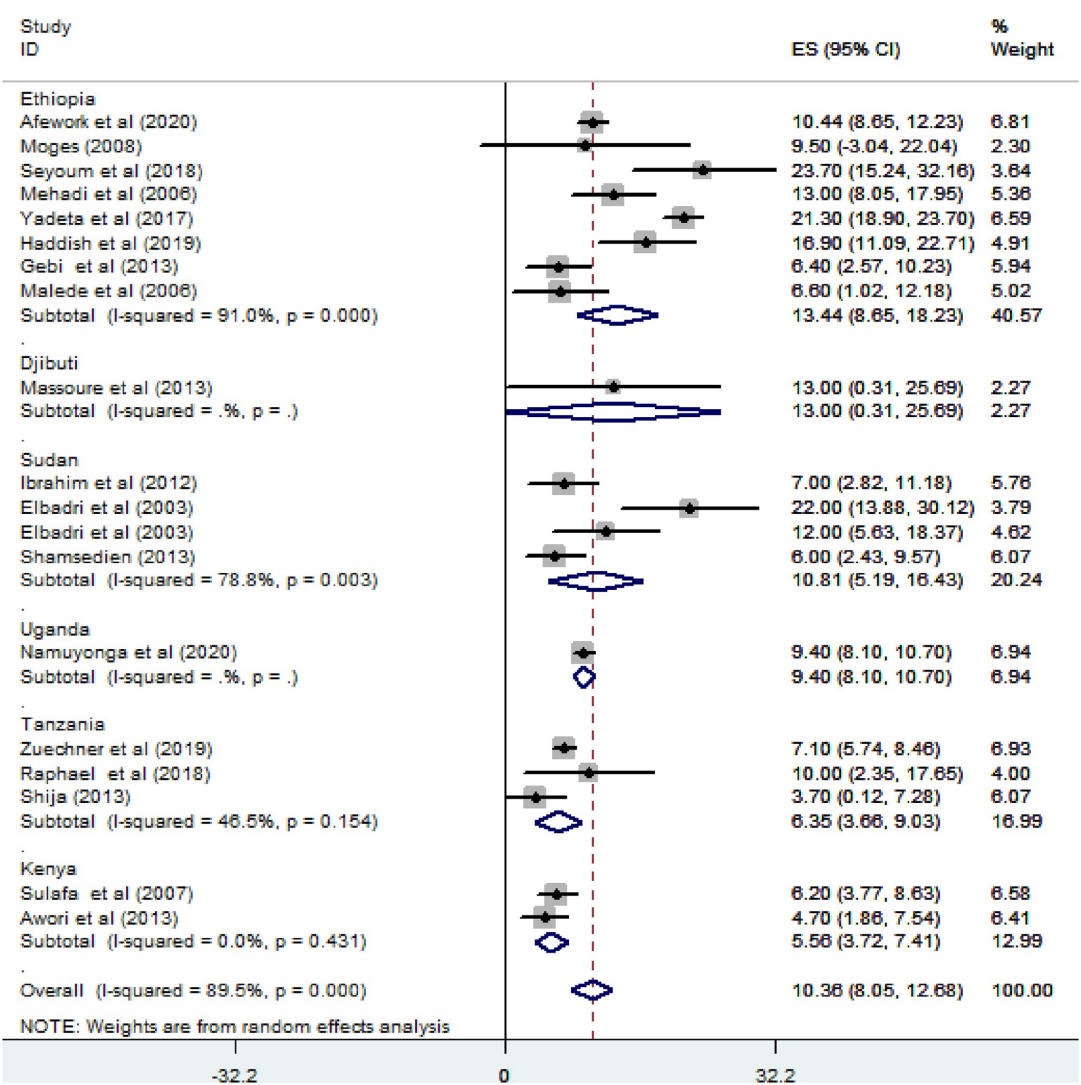
Forest plot showing the subgroup analysis of the prevalence of atrial septal defect among congenital heart defect patients by country in East Africa, from January 2000-October 2020.

**Fig 9:**
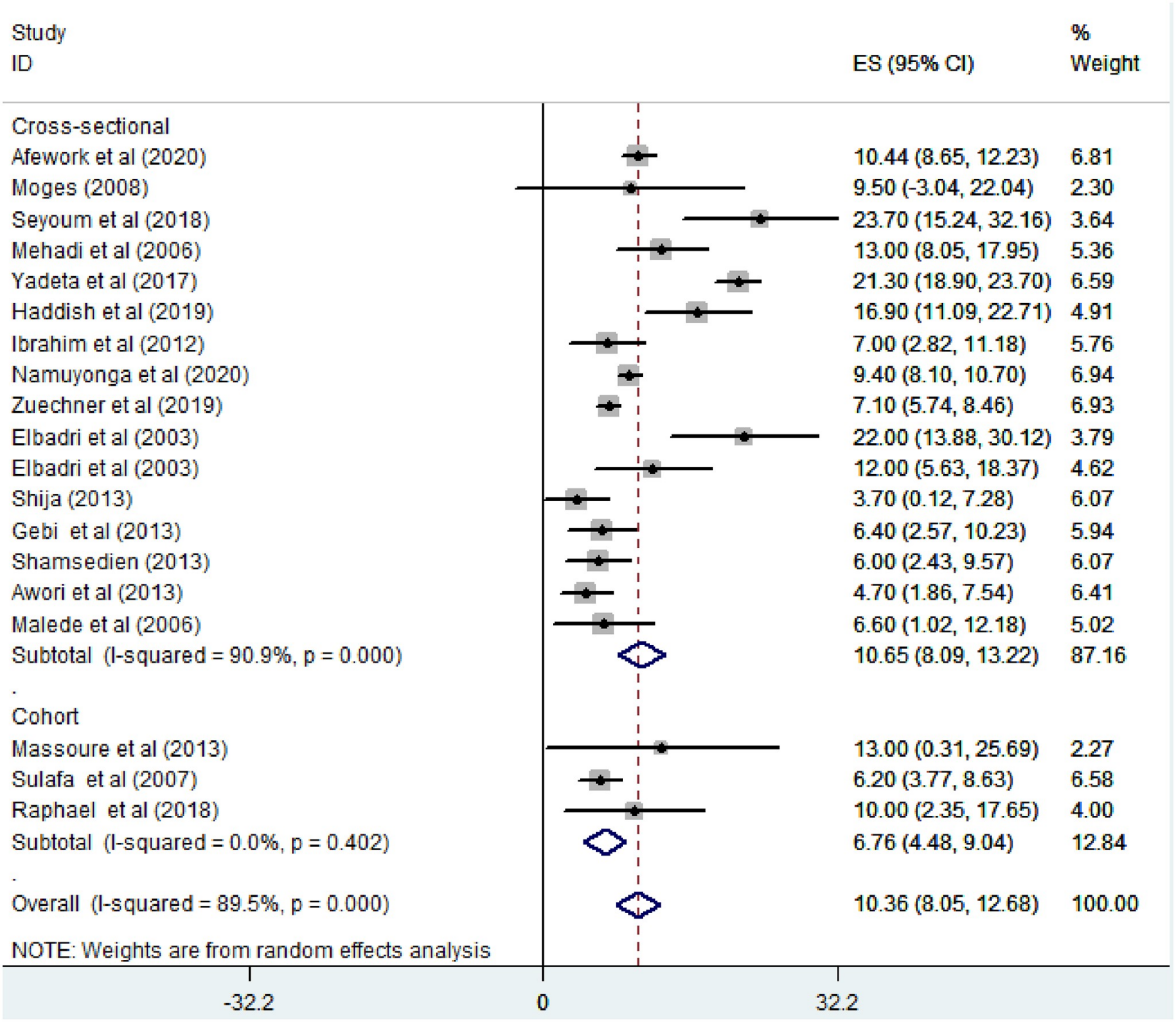
Forest plot showing the subgroup analysis of the prevalence of atrial septal defect among congenital heart defect patients by Study design in East Africa, from January 2000-October 2020.

**Fig 10:**
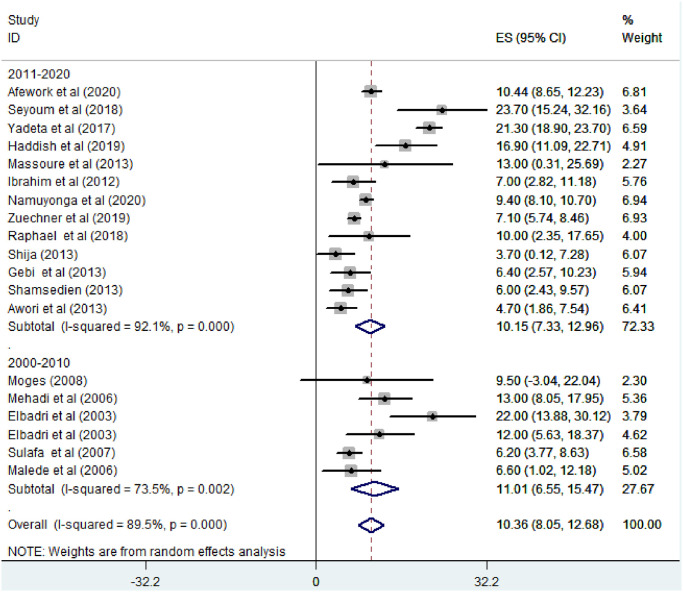
Forest plot showing the subgroup analysis of the prevalence of atrial septal defect among congenital heart defect patients by year of publication in East Africa, from January 2000-October 2020.

*Subgroup analysis for the prevalence of atrial septal defect among congenital heart defect patients in Eastern Africa*. The subgroup analysis was done through stratified by country, study design, and year of publication. Based on this, the prevalence of atrial septal defect among congenital heart defect patients was found 13.44% in Ethiopia, 13% in Djibouti, 10.81% in Sudan, 9.4% in Uganda, 6.35% in Tanzania, and 5.97% in Kenya (Fig 8). Based on the study design, the prevalence of ASD among CHD patients was found to be 10.65% in cross-sectional studies and 6.76% in cohort studies (Fig 9). Based on the year of publication, the prevalence of ASD among CHD patients was found to be 11.01% in studies conducted from January 2000—October 2010 while it was 10.15% where studies conducted from 2011–2020 [Fig 10].

### Heterogeneity

#### Sensitivity analysis for atrial septal defect

We employed a leave-one-out sensitivity analysis to identify the influence of individual studies on the pooled prevalence of atrial septal defect in Eastern Africa. The results of this sensitivity analysis showed that our finding was not dependent on a single study. The pooled estimated prevalence of ASD varied between 9.02 [16] and 10.78 [24] after the deletion of a single study [“S3 Fig”].

### Publication bias

**Fig 11:**
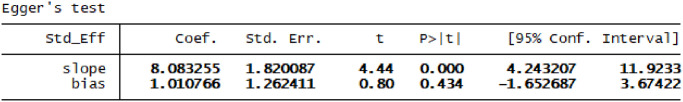
Publication bias on the magnitude of atrial septal defect among congenital heart defect patients in East Africa from January 2000-October, 2020.

A funnel plot showed symmetrical distribution. The Egger’s regression test-value was 0.434, which indicated that, the absence of publication bias [“Fig 11 and S4 Fig”].

## Discussion

Ventricular septal defect and atrial septal defect are the most common form of congenital heart defect and their prevalence is considered to vary significantly with the definition, population, geographical location, and research methods [1]. Therefore, this systematic review and meta-analysis designed to estimate the magnitude of VSD and ASD among CHD patients in the East Africa. Accordingly, the present meta-analyses revealed that, the pooled prevalence of VSD in East Africa is 29.92% (95% CI; 25.84–33.04%). This result was found to be lowered compared to the prevalence of VSD in systematic reviews and meta-analyses conducted globally from the years 1970–2017 and 1930–2010, which was 35.568% (33.876–37.278) [1] and 34% [4] respectively. Similarly, the current prevalence of VSD in the region is still lower compared to other systematic review and meta-analyses conducted in Nigeria in the year 1965–2015, in which VSD accounted for 40.6% (95% CI: 38.9–42.4) in all form of CHDs [32].

The possibility that this low prevalence of VSD in East Africa might be the absence of a complete diagnosis of VSD due to inadequate structured perinatal diagnostic capabilities that potentially leading to a low detection rate. This is also reflected by the paucity of data in the region and the presence of a large pool of older children and adults debilitated with CHD in sub-Saharan Africa [8]. Reversely, developed countries have well-structured perinatal diagnostic facilities that can be diagnosed with a higher number of VSD. Accordingly, studies published from those countries on the prevalence of VSD diagnosed by echocardiography include a relatively high percentage of small defects, however, some of these close spontaneously and many of the remainders cause no clinical problems [5]. Additionally, the difference in the geographical coverage of the studies, the number of studies and/or sample size incorporated and the duration of years considered for their systematic review and meta-analyses could also be contributed to this discrepancy.

Regarding sub-group analysis based on country, the pooled prevalence of VSD in Ethiopia is 36.04%, which is nearly equal to globally conducted meta-analyses and systematic review from 1970–2017, and the year 2011 which was 35.56% [1], and 34% [4] respectively. However, it is lower than meta-analyses and systematic review conducted in Nigeria [32].

The pooled prevalence of ASD in East Africa is 10.36% (95% CI; 8.05–12.68). This result was relatively low compared to the prevalence of ASD in systematic reviews and meta-analyses conducted globally in the years 1970–2017 and 1930–2010, which was 15.37 (95% CI: 13.492–17.363) [1] and 13% [4] respectively. As stated earlier, the possibility that this low prevalence of ASD in East Africa might also, inadequate structured perinatal diagnostic capabilities in the region and reversely true in developed countries, and the difference in the geographical coverage of the studies, the number of studies incorporated and the duration of years considered for their systematic reviews and meta-analyses.

On the other hand, the current prevalence of ASD in the region is comparable with other systematic reviews and meta-analyses conducted in Nigeria in the year 1965–2015, in which ASD accounted for 11.3% (95% CI 10.2–12.5) in all form of CHDs [32]. Regarding the subgroup analysis based on country revealed that, the pooled prevalence of ASD among CHD patients is 13.44% in Ethiopia, 13% in Djibouti, and 9.76% in Sudan. The discrepancies between countries might be due to the number of studies conducted in each country and the emphasis given for the disease, as more emphasis given for the particular disease; more studies can be conducted in the area and better ascertainment of perinatal diagnostic materials and capabilities can be ensured.

Based on the study design, the pooled prevalence of ASD among CHD patients was found to be 10.65% in cross-sectional studies and 6.76% in cohort studies respectively. This might be due to cohort studies apply strict follow-up trends of the patients; through this, they can record more reliable reports of the patients’ overall character and the number of studies conducted in each study design. The pooled prevalence of ASD on the studies conducted from (2000–2010) found to be increased (11.01%) compared to the studies conducted from January 2011–2020 (10.15%). This indicates that ASD is still an alarming issue among CHD patients in East Africa.

Generally, in Eastern regions of Africa, the socioeconomic status of the population and their poor lifestyle will expect a high prevalence of congenital septal defect than developed world. However, the result showed far from the anticipated and the reported prevalence of VSD and ASD in the region continues to be significantly lower than in other regions of the world. This would mislead to underestimate the burden of the disease in the region.

## Conclusions

Based on this review, the pooled prevalence of VSD and ASD is still high and alarming. The result signifies that, the emphasis given for congenital heart disease in East African countries is limited. Therefore, it needs critical interventions at the grass-root level in the region to loosen the burden.

## Recommendations and future implications

Special attention and efforts should be applied for early detection to prevent serious complications and for better prognosis of all forms of CHD to reduce the burden in East Africa. A screening program for CHD should be instituted during the perinatal period. Furthermore, early referral of suspected cases of congenital cardiac anomalies is mandatory for better management till the establishment of cardiac centers in different regions of the continent.

## Supporting information

S1 Fig Sensitivity analysis on the prevalence of ventricular septal defect among CHD patients in East Africa from January 2000-October, 2020. (DOCX)

S2 Fig Funnel plot showing the publication bias on the prevalence of ventricular septal defect among congenital heart defect in East Africa from January 2000-October 2020. (DOCX)

S3 Fig Sensitivity analysis on the prevalence of atrial septal defect among CHD patients in East Africa from January 2000-October, 2020. (DOCX)

S4 Fig Funnel plot showing publication bias on the prevalence of atrial septal defect among congenital heart defect in East Africa from January 2000- October 2020. (DOCX)

S1 Checklist PRISMA 2009 checklist. (DOC)

S1 Data (XLSX)

S2 Data (XLSX)

## Abbreviations

ASD: Atrial Septal Defect
CHD: Congenital Heart Defect
CI: Confidence interval
JBI: Joanna Briggs Institute
PRISMA: Preferred Reporting Items for Systematic Reviews and Meta-Analyses
VSD: Ventricular Septal Defect
