# Supplementary material for: Prevalence of congenital septal defects among congenital heart defect patients in East Africa: A systematic review and meta-analysis
Source: PLoS One. 2021 Apr 22;16(4):e0250006. doi: 10.1371/journal.pone.0250006 (PMC8062078; doi:10.1371/journal.pone.0250006)
Supplement: S3 Fig — (DOCX) [file pone.0250006.s003.docx]

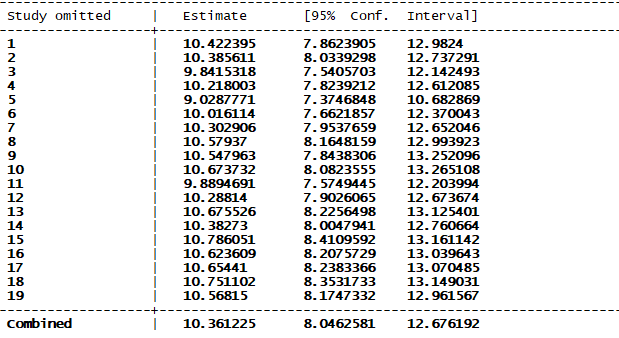


S3 Fig. Shows sensitivity analysis on prevalence of Atrial Septal defect among CHD patients in East Africa from January 2000-October, 2020.
